# Supplementary material for: Individual crop loads provide local control for collective food intake in ant colonies
Source: eLife. 2018 Mar 6;7:e31730. doi: 10.7554/eLife.31730 (PMC5862530; doi:10.7554/eLife.31730)
Supplement: Transparent reporting form [file elife-31730-transrepform.docx]

***eLife’s* transparent reporting form**

We encourage authors to provide detailed information *within their submission* to facilitate the interpretation and replication of experiments. Authors can upload supporting documentation to indicate the use of appropriate reporting guidelines for health-related research (see [EQUATOR Network](http://www.equator-network.org/%20)), life science research (see the [BioSharing Information Resource](https://biosharing.org/)), or the [ARRIVE guidelines](http://www.plosbiology.org/article/info:doi/10.1371/journal.pbio.1000412) for reporting work involving animal research. Where applicable, authors should refer to any relevant reporting standards documents in this form.

If you have any questions, please consult our Journal Policies and/or contact us: [editorial@elifesciences.org](mailto:editorial@elifesciences.org).

**Sample-size estimation**

- You should state whether an appropriate sample size was computed when the study was being designed
- You should state the statistical method of sample size computation and any required assumptions
- If no explicit power analysis was used, you should describe how you decided what sample (replicate) size (number) to use

Please outline where this information can be found within the submission (e.g., sections or figure legends), or explain why this information doesn’t apply to your submission:

The effort to produce errorless identification of local interaction between individual ants required manual recognition process of these interactions. This was the main bottleneck in determining the size of the dataset: Each experiment include more than 1000 interactions and, our main data pool is composed of three replicates of fully analyzed "observation experiments" and two replicates of the "perturbation experiment". The data from these experiments were used to inspect different aspects of the process in study, and the sample size changed accordingly.

The sample size used for each specific analysis can be found in the following figures' captions, table and in the method section:

Fig. 2, Fig. 3, Fig. 4, Fig. 5, Table 1, Fig. S3, Fig. S5, Fig. S6, Methods: Data Analysis: Observation Experiment

**Replicates**

- You should report how often each experiment was performed
- You should include a definition of biological versus technical replication
- The data obtained should be provided and sufficient information should be provided to indicate the number of independent biological and/or technical replicates
- If you encountered any outliers, you should describe how these were handled
- Criteria for exclusion/inclusion of data should be clearly stated
- High-throughput sequence data should be uploaded before submission, with a private link for reviewers provided (these are available from both GEO and ArrayExpress)

Please outline where this information can be found within the submission (e.g., sections or figure legends), or explain why this information doesn’t apply to your submission:

The information regarding the number of replicas is included in the Methods section under 1. Observation Experiment and 2. Perturbation Experiment.

All replications were biological.

Exclusion: Individuals ants that occasionally observed at the food source but clearly did not display foraging cyclic behavior were not considered as foragers (For the specific the criteria See Methods: foragers).

**Statistical reporting**

- Statistical analysis methods should be described and justified
- Raw data should be presented in figures whenever informative to do so (typically when N per group is less than 10)
- For each experiment, you should identify the statistical tests used, exact values of N, definitions of center, methods of multiple test correction, and dispersion and precision measures (e.g., mean, median, SD, SEM, confidence intervals; and, for the major substantive results, a measure of effect size (e.g., Pearson's r, Cohen's d)
- Report exact p-values wherever possible alongside the summary statistics and 95% confidence intervals. These should be reported for all key questions and not only when the p-value is less than 0.05.

Please outline where this information can be found within the submission (e.g., sections or figure legends), or explain why this information doesn’t apply to your submission:

The exact number N (data points) is specified in the legend of figures 2, 3, 4, 5, S3, S4, S5 and inside table 1.

Means and Stand Errors are specified in the legends of figures: 2, 3, 4, 5, S3, S5.

R-Squared is specified in legend of figure 2, in line 523 (for figure S3) and inside the supplementary figure S4.

Pearson's and Spearman's correlation test were used for the data in figure 3 (panels b & c). The results, including p-values, are specified in lines 156 and 158 respectively.

P values of the GLM are presented in table 1 as well the AIC.

(For large datasets, or papers with a very large number of statistical tests, you may upload a single table file with tests, Ns, etc., with reference to sections in the manuscript.)

**Group allocation**

- Indicate how samples were allocated into experimental groups (in the case of clinical studies, please specify allocation to treatment method); if randomization was used, please also state if restricted randomization was applied
- Indicate if masking was used during group allocation, data collection and/or data analysis

Please outline where this information can be found within the submission (e.g., sections or figure legends), or explain why this information doesn’t apply to your submission:

Data point were binned in the following figures 2, 3, 4, 5, S3, S5, S6. The number of points included in the bins are specified in the figures legends.

**Additional data files (“source data”)**

- We encourage you to upload relevant additional data files, such as numerical data that are represented as a graph in a figure, or as a summary table
- Where provided, these should be in the most useful format, and they can be uploaded as “Source data” files linked to a main figure or table
- Include model definition files including the full list of parameters used
- Include code used for data analysis (e.g., R, MatLab)
- Avoid stating that data files are “available upon request”

Please indicate the figures or tables for which source data files have been provided:

The manuscript does not include additional source data.
